# Supplementary material for: The role of CDH2 and MCP-1 mRNAs of blood extracellular vesicles in predicting early-stage diabetic nephropathy
Source: PLoS One. 2022 Apr 1;17(4):e0265619. doi: 10.1371/journal.pone.0265619 (PMC8975111; doi:10.1371/journal.pone.0265619)
Supplement: Attachment — Submitted filename: PONE-D-21-29236.docx [file pone.0265619.s002.docx]

**PONE-D-21-29236**

**Reviewer Response:**

Following amendments and corrections may further improve this manuscript.

1) The manuscript needs a thorough revision regarding its “space” formatting, in all the sections including “Title-statement”.
2) Kindly review, Line and Paragraph-spacing.
3) The manuscript needs a thorough revision for its Grammarly- mistakes; use of helping verbs and tenses.
4) Kindly, add Line numbers, as per journal’s format.
5) Section: Abstract: Methods: Line#4: Kindly abbreviate ROC.
6) Section: Abstract: Results: Line#1: Kindly recorrect the statement as; “The mRNA expression found in CDH2 and MCP, was down-regulated in overt DN group”.
7) Section: Abstract: Results: Line#1: Kindly recorrect as: “0.22- and 0.15-fold change” to “0.22- fold change and 0.15-fold change”, respectively.
8) Section: Abstract: Results: Line#2: Kindly recorrect as: “0.60- and 0.43-fold change” to “0.60- fold change and 0.43-fold change”.
9) Section: Abstract: Results: Line#3: Kindly recorrect as: (1.72- and 2.77-fold change) to (1.72-fold change and 2.77-fold change).
10) Section: Abstract: Results: Line#4: Kindly recorrect as: DM group (0.58-fold change) as compare to control.
11) Section: Abstract: Results: Line#8: (95%CI: 0.65, 0.85) should be written as (95%CI: 0.65-0.85), vice versa throughout the text.
12) Section: Introduction:1^st^ Paragraph: Line 22/23: “while we now know EVs”, should be corrected as “While, now we know that EVs”.
13) It is recommended to correct “mRNAs” throughout the text as “mRNA”, in properly manner.
14) Section: Methods: Subjects and ethical considerations: Line 7 & 9: Kindly correct “creatinineon” as “creatinine on”.
15) Section: Methods: mRNA isolation, cDNA preparation and qRT-PCR analysis: Kindly, add thermo-cycle programming in Tabular-Form;

Initial Denaturation, Denaturation, Annealing, Extension and Final Extension: timing and temperature/ cycle.

16) Section: Results: Demographic information and clinical laboratory characteristics: Line#20: “significant different creatinineconcentration” should be corrected as “significant difference in creatinine concentration”.
17) Section: Results: Diagnostic ability of 1/CDH2 and 1/MCP mRNAs in overt DN detection: Line#1: Kindly, add helping verb as: “discrimination of overt DN from DM”.
18) Section: Results: Diagnostic ability of 1/CDH2 and 1/MCP mRNAs in overt DN detection: Line#3: What do you mean by guaranteed sensitivity=?
19) Section: Results: Diagnostic ability of 1/CDH2 and 1/MCP mRNAs in overt DN detection: Line# 3^rd^ last Line: should be corrected as: ‘for discrimination of overt DN from DM.
20) Section: Discussion: 1^st^ Para: 2^nd^ last Line: “DN progression and develop”, should be corrected as: “DN progression and development”.
21) The manuscript doesn’t include the brief introduction of CDH1, CDH2, MCP & PAI, neither their abbreviation nor their function. No information or Literature Review regarding: How they act as Biomarkers for disease detection, particularly DN? How they help to investigate DN manifestation? Either, they are genes or proteins or their molecular pathway is associated with DN related Risk Factors? Need to Elaborate exclusively.
