# Supplementary material for: The role of CDH2 and MCP-1 mRNAs of blood extracellular vesicles in predicting early-stage diabetic nephropathy
Source: PLoS One. 2022 Apr 1;17(4):e0265619. doi: 10.1371/journal.pone.0265619 (PMC8975111; doi:10.1371/journal.pone.0265619)
Supplement: Attachment — Submitted filename: Response to Reviewers.docx [file pone.0265619.s003.docx]

Response to Reviewers

Reviewer #1: Reviewer Response:

Following amendments and corrections may further improve this manuscript.

1) The manuscript needs a thorough revision regarding its “space” formatting, in all the sections including “Title-statement”.

Done, you are right. Thanks for your appropriate comment. We correct “space” formatting of all part of the text in the revised manuscript.

2) Kindly review, Line and Paragraph-spacing.

Done, Thanks for your comment. Manuscript text was double-spaced.

3) The manuscript needs a thorough revision for its Grammarly- mistakes; use of helping verbs and tenses.

Done, Thanks for your precise comment. We corrected the Grammarly- mistakes to the best of our knowledge.

4) Kindly, add Line numbers, as per journal’s format.

Done, you are right. We added continuous Line numbers.

5) Section: Abstract: Methods: Line#4: Kindly abbreviate ROC.

Done, you are right. We wrote the full name of ROC in this part of revised manuscript.

6) Section: Abstract: Results: Line#1: Kindly recorrect the statement as; “The mRNA expression found in CDH2 and MCP, was down-regulated in overt DN group”.

Done, Thanks for your excellent comment. We recorrect this statement in the abstract section of revised manuscript.

7) Section: Abstract: Results: Line#1: Kindly recorrect as: “0.22- and 0.15-fold change” to “0.22- fold change and 0.15-fold change”, respectively.

Done, Thanks for your precise comment. We recorrected this statement in the revised manuscript.

8) Section: Abstract: Results: Line#2: Kindly recorrect as: “0.60- and 0.43-fold change” to “0.60- fold change and 0.43-fold change”.

Done, Thanks for your precise comment. We recorrected this statement in the revised manuscript.

9) Section: Abstract: Results: Line#3: Kindly recorrect as: (1.72- and 2.77-fold change) to (1.72-fold change and 2.77-fold change).

Done, Thanks for your comment. We recorrected this statement in the revised manuscript.

10) Section: Abstract: Results: Line#4: Kindly recorrect as: DM group (0.58-fold change) as compare to control.

Done, Thanks for your appropriate comment. We recorrected it in the revised manuscript.

11) Section: Abstract: Results: Line#8: (95%CI: 0.65, 0.85) should be written as (95%CI: 0.65-0.85), vice versa throughout the text.

Done, Thanks for your promising comment. We recorrect this issue throughout the text.

12) Section: Introduction:1st Paragraph: Line 22/23: “while we now know EVs”, should be corrected as “While, now we know that EVs”.

Done, Thanks a lot for your precise comment. We recorrect this statement.

13) It is recommended to correct “mRNAs” throughout the text as “mRNA”, in properly manner.

Done, thanks for your promising comment. We correct “mRNAs” as “mRNA” throughout the text in proper manner.

14) Section: Methods: Subjects and ethical considerations: Line 7 & 9: Kindly correct “creatinineon” as “creatinine on”.

Done, you are right. We correct it. Besides, we revised the text in the manner of “spacing”.

15) Section: Methods: mRNA isolation, cDNA preparation and qRT-PCR analysis: Kindly, add thermo-cycle programming in Tabular-Form;

Initial Denaturation, Denaturation, Annealing, Extension and Final Extension: timing and temperature/ cycle.

Done, Thanks for your comment.

16) Section: Results: Demographic information and clinical laboratory characteristics: Line#20: “significant different creatinineconcentration” should be corrected as “significant difference in creatinine concentration”.

Done, Thanks for your comment. We correct this.

17) Section: Results: Diagnostic ability of 1/CDH2 and 1/MCP mRNAs in overt DN detection: Line#1: Kindly, add helping verb as: “discrimination of overt DN from DM”.

Done, Thanks for your precise comment. We corrected it in the revised manuscript.

18) Section: Results: Diagnostic ability of 1/CDH2 and 1/MCP mRNAs in overt DN detection: Line#3: What do you mean by guaranteed sensitivity=?

Done, you are right. This sentence was vague; so we replaced it with “Based on Youden's J statistic, 1/CDH2 mRNA had optimal diagnostic performance at 2.14-fold change. In this cutoff, 1/CDH2 mRNA had sensitivity of 74.3%, specificity of 69.4%, PPV of 57.8%, and NPV of 82.7% for overt DN detection.”. In this sentence, we reported the sensitivity, specificity, PPV, and NPV of 1/CDH2 mRNA at cutoff of 2.14-fold change.

19) Section: Results: Diagnostic ability of 1/CDH2 and 1/MCP mRNAs in overt DN detection: Line# 3rd last Line: should be corrected as: ‘for discrimination of overt DN from DM.

Done, Thanks for your precise comment. We corrected it in the revised manuscript.

20) Section: Discussion: 1st Para: 2nd last Line: “DN progression and develop”, should be corrected as: “DN progression and development”.

Done, Thanks for your precise comment. We corrected it.

21) The manuscript doesn’t include the brief introduction of CDH1, CDH2, MCP & PAI, neither their abbreviation nor their function. No information or Literature Review regarding: How they act as Biomarkers for disease detection, particularly DN? How they help to investigate DN manifestation? Either, they are genes or proteins or their molecular pathway is associated with DN related Risk Factors? Need to Elaborate exclusively.

Done, Thanks for your precise comment. We wrote the full name of the investigated genes and their abbreviations. We explained the productions and function of the investigated genes. We explained the pathological conditions related to the abnormal expression of the investigated genes. We provided the literature review regarding the potential ability of the investigated mRNAs (the transcript of investigated genes) that act as Biomarkers for renal fibrosis detection, especially due to DN. We wrote the usefulness of the investigated mRNAs for DN manifestations including “these biomarkers can accurately detect DN patients in its early-stage, stratify them into different stages for individualized management, and monitor their response to therapy”. We presented that the expression mRNA levels of the investigated genes were evaluated to identify whether they were associated with DN or not.

Introduction section; page 3; lines 136-139

Introduction section; page 4; lines 164-170

Introduction section; page 5; lines 170-190

22) Kindly also provide with brief explanation of Biomarkers and how they help to investigate disease progression?

Done, Thanks for your excellent comment. We explained the usefulness of new biomarkers, especially when combined with currently used biomarkers, for detecting, stratifying, and monitoring disease progression in the introduction section of revised manuscript. Introduction section; page 3; lines 136-139

Reviewer #2: Comments

The authors addressed diabetic nephropathy (DN) patients in their study. They studied the expression of a panel of DN related genes in blood extracellular vesicles (EVs) of 4 groups; overt DN patients, incipient DN patients, diabetes mellitus patients (DM), and healthy controls. They reported that CDH2 and MCP mRNA expression is significantly downregulated in blood EVs of overt and incipient DN patients relative to DM patients lacking DN. CDH2 gene was found more downregulated in EVs of overt DN patients compared to incipient and DM patients. Expression of both genes was inversely correlated with serum creatinine levels and degree of albuminuria. They suggested that CDH2 and MCP-1 mRNA expression declines as DN develops, indicating a possible renoprotective effect of these mRNAs in diabetic individuals and their possible role as diagnostic biomarkers for early-stage DN that may allow monitoring its progression. The statistical studies are very good. The study is interesting and concerns a considerable number of researchers and physicians.

1) The full names of the investigated genes should be written in the introduction with a referral to their functions.

Done, you are right. Thanks for your appropriate comment. We added the full names of the investigated genes as well as their functions in the introduction section of the revised manuscript.

Introduction section; page 4; lines 164-170

Introduction section; page 5; lines 170-188

2) In the methods section, Subjects and ethical considerations sub-section, please, preferably mention the type of diabetes mellitus from which the samples were obtained. Which type of DM was addressed? or were both types included in the study?

Done, Thanks for your suitable comment. All participants had type 2 diabetes mellitus and we mentioned it in the method section of revised manuscript.

3) In the methods section, Sample collection and clinical laboratory tests section, please, put a reference for the Cockcroft–Gault Equation used in this section.

Done, Thanks for your comment. We added the reference for Cockcroft–Gault Equation in the revised manuscript.

4) In the methods section, mRNA isolation, cDNA preparation and qRT-PCR analysis sub-section:

- “TRIzolTM Reagent(USA, Cat. Num: 15596026. Germany)”: please, mention the company and revise the manufacturer’s country.

-“ RevertAid First Strand cDNA Synthesis Kit (Fermentas, Cat Num: K1622, USA), Is it Fermentas or Thermo Scientific?

- “Nanodrop (Thermo Scientific 2000)”- “SYBR Green PCR Master Mix (Applied Biosystems)”: please mention the country.

- Were the used primers designed by authors or obtained from previous studies? , if so, please, mention the reference(s).

Done, Thanks for your comment.

5) In the Results section, Demographic information and clinical laboratory characteristics subsection:

- In table 1, does the p value represent the difference between the 4 groups?

Yes, you are right. This p value represents the difference between the 4 groups.

It will be better to put the p value of the pairwise comparison (the difference between each 2 groups) by subdividing the p value column so that the results become clearer.

Done, Thanks for your appropriate comment. We did the pairwise comparisons of parameters that were significantly different between the 4 groups in Table 1 and we showed the pairwise comparisons in Table 2.

- For the creatinin concentration results, in the following part: “Incipient DN group had higher creatinine concentration than DM group (p = 0.001) and no significant different creatinine concentration compared to control (p = 0.151).”

Do you mean no significant difference between DN group and control group or between DM group and control group? Please, clarify.

Done, you are right. This statement was vague. We mean no significant difference between incipient DN group and control group. We clarified this finding in the revised manuscript.

6) In the Results section, Characterization of blood Evs subsection, in the following part:

“Figure 1 showed the results of the size and morphology of isolated exosomes which were analyzed by SEM and DLS. The results revealed that size of isolated EVs were between 80-150 nm”, you mentioned that the isolated EVs are exosomes, however the size stated was “80-150 nm” which means that not all the isolated EVs are exosomes according to the classification mentioned in the introduction: “Exosomes are 30–100 nm in diameter, derived from the endosomal pathway and fusion of multivesicular bodies to plasma membranes, while microvesicles, with a diameter of 100-1,000 nm, are derived from budding and evagination of plasma membranes.(16)”

Done, you are absolutely right. The isolated EVs were both exosomes and microvesicles. Therefore, we revised the manuscript and in every statement that we wrongly stated exosomes, we replaced them with extracellular vesicle (EV).

-Figure 1a shows an EV with a diameter of 152 nm so the size range would be: (80-152 nm) to be more precise.

Done, you are right. We corrected it in the revised manuscript.

7) In the Results section, mRNAs expression level in blood EVs subsection:

-In table 2, it is better to present the p values of the pairwise comparison (presented later in Table S 1 with no referral to this table in the text), as these results are important and should be presented in the main table (Table 2) not in a supplementary one.

Done, you are right. Thanks for your appropriate comment. We presented Table S1 as Table 4 in the main text.
