# Supplementary material for: The role of CDH2 and MCP-1 mRNAs of blood extracellular vesicles in predicting early-stage diabetic nephropathy
Source: PLoS One. 2022 Apr 1;17(4):e0265619. doi: 10.1371/journal.pone.0265619 (PMC8975111; doi:10.1371/journal.pone.0265619)
Supplement: Attachment — Submitted filename: Comments2.docx [file pone.0265619.s004.docx]

Comments

I am satisfied with the response and the revision of authors, however, I suggest to make a couple of corrections:
1. As authors’ results did not indicate a role for CH1 and PAI mRNA of blood EVs in early detection of DN, would it be better to modify the title of the manuscript to be more relevant to the results?

2. I would recommend some close proofreading; some punctuation still needed, and the reference number should be placed in the end of the sentence before the full stop not after.
