# Supplementary material for: The role of CDH2 and MCP-1 mRNAs of blood extracellular vesicles in predicting early-stage diabetic nephropathy
Source: PLoS One. 2022 Apr 1;17(4):e0265619. doi: 10.1371/journal.pone.0265619 (PMC8975111; doi:10.1371/journal.pone.0265619)
Supplement: Attachment — Submitted filename: Response to Reviewers (4).docx [file pone.0265619.s006.docx]

Response to Reviewers:

Additional Editor Comments:

There are several structural, grammatical, punctuation, and capitalization errors that require heavy editing by a native English speaker. Please revise the organization of the tables as there are supplementary tables uploaded but not indicated in the text.

Done, Thanks for your comment. A native English speaker has heavily edited the final version of this manuscript in terms of structural, grammatical, punctuation, and capitalization errors. We also organized all the tables in the revised manuscript. In this case, we had 8 tables in the revised manuscript that were cited in ascending numeric order upon first appearance in the manuscript file from Table 1 to Table 8. Besides, we provided the raw data of participants as S1 Appendix.
